# SG-APSIC1121: Family caregivers in the patient room: Exploring the family involvement in care provision across hospital settings from an infection prevention and control (IPC) perspective

**DOI:** 10.1017/ash.2023.64

**Published:** 2023-03-16

**Authors:** Ji yeon Park, Holly Seale, Jerico Franciscus Pardosi

**Affiliations:** University of New South Wales, Sydney, Australia; University of New South Wales, Sydney, Australia; Queensland University of Technology, Brisbane, Australia

## Abstract

**Objectives:** Across many Asian countries, family caregivers provide a wide range of patient care activities while staying in the patient’s room. This unique care arrangement has been reported as a factor contributing to the spread of outbreaks including Middle East respiratory syndrome and coronavirus disease 2019 (COVID-19) in many Asian countries. We sought to understand the context in which direct patient care activities are provided by family caregivers and/or private caregivers in hospitals across Bangladesh, Indonesia, and South Korea from the infection prevention and control (IPC) perspective. **Methods:** We used a multimethod design with both quantitative and qualitative approaches. In total, 432 patients were surveyed from 5 tertiary-care hospitals across 3 selected countries, and 64 participants from 2 groups were interviewed: group A comprised patients, family caregivers and private caregivers and group B comprised healthcare workers. Survey data were analyzed descriptively, and the interview data were analyzed using thematic analysis. **Results:** The study findings highlight the different landscapes of care provision in the selected countries. Both the interviews and surveys highlighted 2 aspects of family caregiving. (1) Family caregivers inhabit in the patient zone for long periods, resulting in overcrowding, and (2) they provide a wide ranges of physically associated care activities, including those associated with the risk of healthcare-associated infections (HAIs). Despite the high number of family caregivers and their in-depth involvement in direct patient care, education and support provided to family caregivers around IPC/HAI were insufficient and varied. Also, challenges related to maintaining adequate hygiene in the environment for minimum IPC were reported. **Conclusions:** This study has elucidated the current landscape of family involvement in inpatient care provision and acknowledges their contribution to high risks of HAI, as well as their current lack of IPC knowledge and practice. These findings reveal that future updates in IPC strategy should acknowledge this arrangement with family caregivers and should address this role with IPC measures.

